# Incidence, risk factors and prognosis of acute kidney injury in patients treated with immune checkpoint inhibitors: a retrospective study

**DOI:** 10.1038/s41598-022-21912-y

**Published:** 2022-11-05

**Authors:** Ming-Su Ji, Rilige Wu, Zhe Feng, Yuan-Da Wang, Yong Wang, Li Zhang, Xue-Feng Sun, Xiang-Mei Chen, Kun-Lun He, Guang-Yan Cai

**Affiliations:** 1 Department of Nephropathy, The First Medical Center of Chinese PLA General Hospital, Beijing, 100853 China; 2 Medical Big Data Research Center, Chinese PLA General Hospital, Beijing, 100853 China

**Keywords:** Nephrology, Oncology

## Abstract

Immune checkpoint inhibitors (ICIs) change the prognosis of many cancer patients. With the increasing use of ICIs, immune-related adverse events are occurring, including acute kidney injury (AKI). This study aimed to assess the incidence of AKI during ICI treatment and its risk factors and impact on mortality. Patients treated with ICIs at the First Medical Center of the Chinese PLA General Hospital from January 1, 2014, to December 30, 2019, were consecutively enrolled, and risk factors affecting AKI development in patients treated with ICIs were analyzed using univariate and multivariate logistic regression. Medical record surveys and telephone inquiry were used for follow-up, and Kaplan–Meier survival analysis and Cox regression were used to analyze independent risk factors for death. Among 1615 patients, 114 (7.1%) had AKI. Multivariate logistic regression analysis showed that anemia, Alb < 30 g/L, antibiotic use, diuretic use, NSAID use and proton pump inhibitor use were independent risk factors for AKI development in patients treated with ICIs. Stage 2 or 3 AKI was an independent risk factor for nonrecovery of renal function after AKI onset. Multivariate Cox regression analysis showed that anemia, Alb < 30 g/L, AKI occurrence, and diuretic use were independent risk factors for death in patients treated with ICIs, while high baseline BMI, other tumor types, ACEI/ARB use, and chemotherapy use were protective factors for patient death. AKI occurs in 7.1% of patients treated with ICIs. Anemia, Alb < 30 g/L, and combined medication use are independent risk factors for AKI in patients treated with ICIs. Anemia, Alb < 30 g/L, AKI occurrence, and diuretic use were independent risk factors for death in patients treated with ICIs.

## Induction

The recent development of immune checkpoint inhibitors (ICIs) has driven a revolutionary change in cancer treatment^1^. ICIs target immune effector cells, primarily T cells, which can result in an effective immune response against tumor cells^2^. This new treatment improves the outcomes of human cancer patients, and it is now widely used in an increasing number of tumor types^3^, such as melanoma, lung cancer, and urothelial and renal cell carcinomas. ICIs act by targeting several immune checkpoints, including cytotoxic T lymphocyte–associated antigen 4 (CTLA-4), programmed cell death protein 1 (PD-1), and programmed death-ligand 1 (PD-L1). However, ICIs have side effects, such as colitis, hypophysitis, rash, pneumonitis, hypothyroidism, arthralgia and vitiligo, which are called immune-related adverse events (irAEs)^4^. This treatment may also bring about nephrotoxicity. Renal toxicity from ICIs is not common in clinical trials^5–11^. However, in recent years, irAEs in the kidney have received increasing attention, and a series of renal irAEs have been described in case reports and case series, including acute interstitial nephritis (AIN)^12^, acute tubular necrosis (ATN)^13^, and glomerular disease^14^.

Acute kidney injury (AKI) is a clinical syndrome characterized by the deterioration of renal function that rapidly develops over hours to days^15^. The acute phase of AKI not only causes disturbances in the internal environment and increases the risk of death but also has important long-term effects on multiple organs, such as the heart, lungs, and brain. AKI is also an important factor in prolonging patients' hospital stays, increasing long-term and short-term mortality, and increasing their hospital costs^16^. AKI associated with ICIs is rare in clinical trials^5–11^, but its incidence in real life may be higher than previously reported. Previously, it was reported in the literature that the incidence of ICI-AKI was 1.4 to 4.9%^17,18^. In the past two years, with the increasing use of ICIs, recent studies on ICI-AKI have shown that the incidence of ICI-AKI is approximately 14–18%^19–23^.

Studies have shown that age and baseline renal function are independent risk factors for the occurrence of ICI-induced AKI^23^. A multicenter study showed that the use of proton pump inhibitors (PPIs) and extrarenal irAEs are both associated with a higher risk of ICI-AKI^24^. In addition, whether AKI is associated with an increased risk of death is still inconclusive. Research by Alejandro Meraz-Muñoz et al. showed that AKI was not associated with death^19^, but in the study of Frank B. Cortazar et al., if renal function does not recover in patients with ICI-AKI, it is independently associated with a higher mortality rate^25^. The study by Clara Garcıá-Carro et al. also found that AKI was a risk factor for death^23^.

However, there are still relatively few data from studies on AKI during ICI treatment, especially in Asian patients. Given that China has a large cancer population, an increasing number of patients will be treated with ICIs. Our goal is to clarify the incidence and risk factors of AKI during ICI treatment and its effect on mortality in Chinese patients.

## Methods

### Study design and patient selection

**Figure 1:**
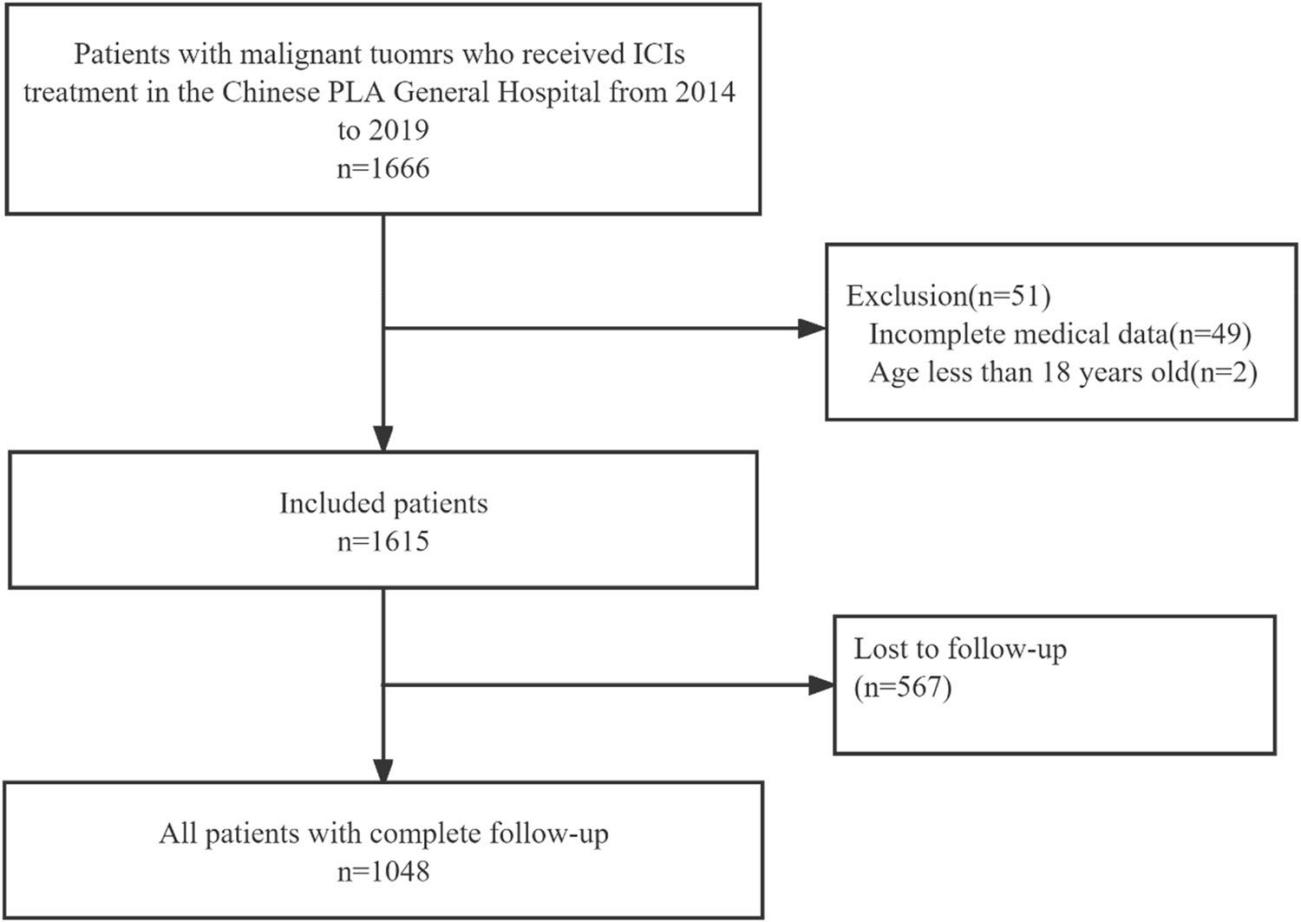
Flow chart of this study.

A retrospective study was performed on 1666 patients treated with ICIs who were hospitalized at the Chinese PLA General Hospital from January 2014 to December 2019. The inclusion criteria were as follows: (1) patients were aged ≥ 18 years; (2) patients admitted to the hospital were mainly diagnosed with solid malignant tumors; and (3) patients were hospitalized in our hospital and received at least one dose of the following ICIs: nivolumab, pembrolizumab, atezolizumab, durvalumab, sintilimab, camrelizumab, toripalimab and ipilimumab. The exclusion criteria were as follows: (1) patients with end-stage renal disease (ESRD); (2) patients with a history of nephrectomy or kidney transplantation; and (3) patients with incomplete data during hospitalization and follow-up. Two patients were excluded because they were younger than 18 years, and 49 patients were excluded because they had incomplete medical data (Fig. 1).

### Outcome

The primary outcome was the incidence of AKI during treatment with ICIs. AKI was identified and staged by fold-change in creatinine from baseline according to Kidney Disease Improving Global Outcomes (KDIGO) AKI criteria within 12 months of checkpoint inhibitor initiation^26,27^. Urinary volume was not used as a diagnostic criterion in this study because the measurement of urine volume is influenced by several factors. This study focused on the first episode of AKI after the administration of ICIs. We analyzed the independent risk factors affecting the occurrence of AKI in patients treated with ICIs. We analyzed the independent risk factors for failure to recover renal function in patients with AKI treated with ICIs, and recovery of renal function was defined as recovery of serum creatinine (sCr) to < 1.5 times the baseline value within 90 days after AKI. Because we wanted to examine whether the inability to recover renal function within 90 days after the onset of AKI affected the patient's mortality, we removed the missing values without prognostic follow-up results for the accuracy of the results and did not interpolate.

We also compared mortality in patients with AKI and those without AKI, analyzing risk factors affecting mortality in patients receiving ICIs. The study started follow-up from the time of patient enrollment using a combination of hospital electronic medical records and telephone inquiries, with the endpoint event of patient death or survival and recording the patient's date of death. The enrollment date was the first day of ICI administration. The follow-up cutoff date was September 1, 2021.We lost some data during this 5-year period because many patients treated with ICIs refused to respond to follow-up calls. The follow-up rate was 64.9% (1048/1615). This investigation involved informed consents from all individual participants as well as ethical approval granted by the Ethics Committee in the Chinese PLA General Hospital (Approval No. 2013–050-01).

### Clinical collection and definitions

Clinical data were collected from patients treated with ICIs, including age, gender, body mass index (BMI), tumor type, concomitant medications and comorbidities. The estimated glomerular filtration rate (eGFR) was calculated using the eGFR-EPI formula^28^. Chemotherapeutic medicine included cisplatin, carboplatin, mitomycin, isocyclophosphamide, pemetrexed, cetuximab, methotrexate, and colchicine. Antibiotic medicine included aminoglycoside antibiotics, vancomycin, amphotericin B, rifampin, ciprofloxacin, and sulfonamide antibiotics. The baseline collection time was the baseline value measured closest to the dosing date before the patient first received ICIs, and concomitant medication was the dosing status during the patient's treatment with ICIs. ESRD was defined as eGFR ≤ 15 mL/min/1.73 m^2^ or the need for renal replacement therapy (RRT). Extrarenal irAEs were recorded based on diagnostic codes and medical records, with concomitant occurrence of AKI. Treatment for AKI and overall outcomes, including survival during follow-up, were documented. All AKI cases were reviewed by two nephrologists, and the causes of AKI were divided into four categories: checkpoint inhibitor-associated AKI, hemodynamic AKI/acute tubular necrosis (ATN), obstructive AKI, and other causes of AKI. Potential checkpoint inhibitor-associated AKI was defined in patients who, after evaluation by two nephrologists, showed no evidence of another possible cause of AKI and were experiencing another irAE^20,27^. Hemodynamic AKI/ATN included AKI occurring in the setting of dehydration (circulatory collapse, diarrhea, vomiting, etc.), septic or ischemic ATN conditions, and obstructive AKI included all identified causes of bilateral ureteral or urethral orifice obstruction.

### Statistical analysis

SPSS 24.0 software was used for statistical analysis. Normally distributed measures are expressed as $\overline{\rm X}\pm {\rm s}$, and independent samples t test was used for comparison between groups; count data are expressed as number of cases and percentages, and χ2 test was used for comparison between groups. Univariate and multivariate logistic regression analyses were used to identify AKI risk factors; the independent variables included in the logistic regression analysis were selected based on the results of previous studies^19,25,27,29,30^. We report the odds ratios (ORs) with 95% confidence intervals (CIs) for each covariate of interest. Variables with P values < 0.05 in univariate analysis were selected as independent variables for multivariate analysis. Multivariate logistic regression was used to determine the predictors of renal recovery in patients with ICI-AKI, and the independent variables included in the logistic regression analysis were selected based on the results of previous studies^25,30^.

Kaplan–Meier curves and multivariate Cox regression models were used to assess the relationship between the occurrence of AKI and the recovery of renal function after the onset of AKI and other factors affecting survival. Hazard ratios (HR) and 95% CIs for mortality were reported. Two-sided *P* values < 0.05 were considered statistically significant.

### Statement

We confirm that all methods were carried out in accordance with relevant guidelines and regulations in methods section.

## Results

### Characteristics of the study cohort

**Figure 2:**
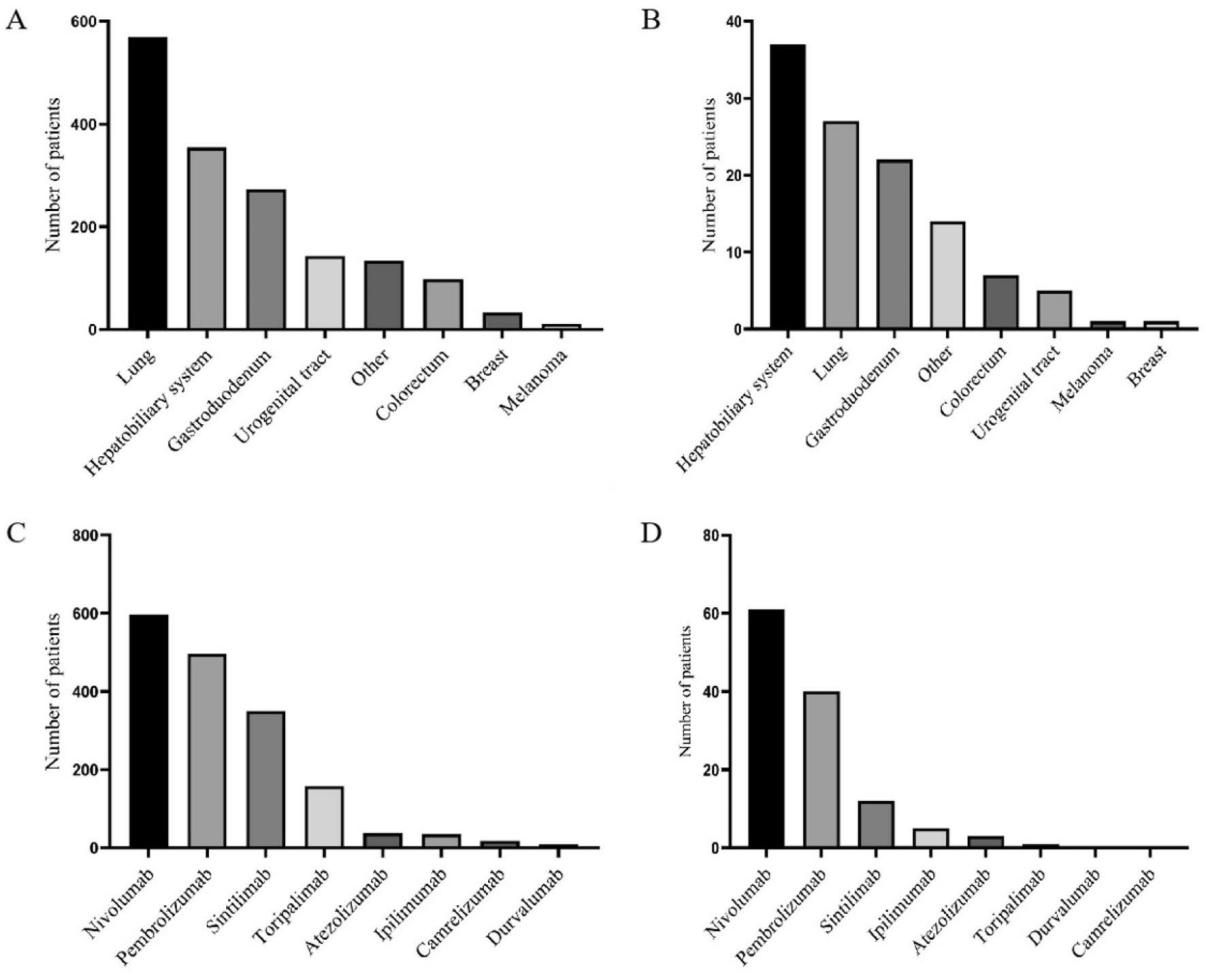
Distribution of tumor types in patients treated with ICIs (**A**). Distribution of tumor types in patients with AKI after treatment with ICIs (**B**). Distribution of ICI usage (**C**). Distribution of ICI use in AKI patients (**D**).

During the study period, 1615 patients were treated with ICIs at our center and were screened for inclusion in the study. The average age was 57.41 ± 11.59 years; among them, 1115 patients (69.0%) were male, 569 patients (35.2%) had lung cancer, and 354 patients (21.9%) had tumors of the hepatobiliary system (Fig. 2). Anti-PD-1 was the most common type of ICI used (96.7% of patients) (Fig. 2). The baseline sCr level was 70.74 ± 23.61 μmol/L, and the median follow-up time was 26.8 months (Table 1).

**Table 1:** Baseline characteristics and univariate analysis comparing AKI and non-AKI patients.

Variable	Cohort	AKI	Non-AKI	*P* value
n	1615	114	1501	
Age (years)	57.41 ± 11.59	56.52 ± 11.46	57.48 ± 11.60	0.391
Male, n (%)	1115 (69.0)	76 (66.7)	1039 (69.2)	0.643
Baseline creatinine (μmol/L)	70.74 ± 23.61	66.55 ± 22.79	71.05 ± 23.65	0.049
Baseline eGFR (mL/min•1.73m^2^)	94.96 ± 19.40	98.50 ± 20.91	94.69 ± 19.26	0.043
BMI (kg/m^2^)	23.02 ± 3.63	22.43 ± 3.79	23.06 ± 3.61	0.078
**Baseline Alb, n (%)**				< 0.001
< 30 g/L	71(4.4)	10(8.8)	61(4.1)	
30 g/L ~ 35 g/L	336(20.8)	43(37.7)	293(19.5)	
> 35 g/L	1208(74.8)	61(53.5)	1147(76.4)	
**Malignancy, n (%)**				0.016
Breast	33(2.0)	1(0.9)	32(2.1)	
Colorectum	98(6.1)	7(6.1)	91(6.1)	
Gastroduodenum	273(16.9)	22(19.3)	251(16.7)	
Urogenital tract	143(8.9)	5(4.4)	138(9.2)	
Hepatobiliary system	354(21.9)	37(32.5)	317(21.1)	
Lung	569(35.2)	27(23.7)	542(36.1)	
Melanoma	11(0.7)	1(0.9)	10(0.7)	
Other	134(8.3)	14(12.3)	120(8.0)	
**Comorbidities, n (%)**				
Cerebrovascular disease	50 (3.1)	3 (2.6)	47 (3.1)	0.987
CHD	118 (7.3)	9 (7.9)	109 (7.3)	0.949
Diabetes	272 (16.8)	20 (17.5)	252 (16.8)	0.938
Hypertension	439 (27.2)	32(28.1)	407 (27.1)	0.911
Liver disease	228 (14.1)	23 (20.2)	205 (13.7)	0.074
Anemia	993(61.5)	93(81.6)	900(60.0)	< 0.001
CKD	102(6.3)	9(7.9)	93(6.2)	0.604
Autoimmune disease	5(0.3)	1(0.9)	4(0.3)	0.797
**Concomitant medications, n (%)**				
ACEI/ARB	206 (12.8)	3 (2.6)	203 (13.5)	0.001
PPI	1058 (65.5)	97 (85.1)	961 (64.0)	< 0.001
Diuretics	303 (18.8)	43 (37.7)	260 (17.3)	< 0.001
NSAIDs	590 (36.5)	84 (73.7)	506 (33.7)	< 0.001
Antibiotics	34 (2.1)	10(8.8)	24 (1.6)	< 0.001
Chemotherapy	367 (22.7)	24 (21.1)	343 (22.9)	0.744
**Immunotherapy, n (%)**				
Ipilimumab	35 (2.2)	5 (4.4)	30 (2.0)	0.176
Nivolumab	597 (37.0)	61 (53.5)	536 (35.7)	< 0.001
Pembrolizumab	497 (30.8)	40 (35.1)	457 (30.4)	0.352
Durvalumab	9 (0.6)	0 (0.0)	9 (0.6)	0.86
Toripalimab	158(9.8)	1(0.9)	157(10.5)	0.002
Sintilimab	349(21.6)	12(10.5)	337(22.5)	0.004
Camrelizumab	18(1.1)	0(0.0)	18(1.2)	0.476
Atezolizumab	37(2.3)	3(2.6)	34(2.3)	1
Extrarenal irAEs	46(2.8)	7(6.1)	39(2.6)	0.057
ICI class, n (%)				
Anti-PD-1	1561 (96.7)	110(96.5)	1451 (96.7)	1
Anti-PD-L1	46 (2.8)	3(2.6)	43 (2.9)	1
Anti-CTLA-4	35 (2.2)	5 (4.4)	30 (2.0)	0.176
AKI stage, n (%)				
Stage 1		87(76.3)		
Stage 2		19(16.7)		
Stage 3		8(7.0)		
**Etiology among patients with AKI**				
Potential ICI-related AKI		6(5.2)		
Hemodynamic AKI/ATN		35(30.7)		
Obstructive AKI,		11(9.6)		
Other causes of AKI		62(54.4)		

*ACEI* angiotensin-converting enzyme inhibitor, *ARB* angiotensin receptor blocker, *AKI* acute kidney injury, *eGFR* estimated glomerular filtration rate, *CHD* coronary heart disease, *NSAIDs *nonsteroidal anti-inflammatory drugs, *PPI* proton pump inhibitor, CTLA-4 cytotoxic T lymphocyte–associated antigen 4, PD-1 programmed cell death 1, PD-L1 programmed death-ligand 1, *BMI*, body mass index, *Alb* albumin.

### Baseline characteristics of patients in the AKI and non-AKI groups

A total of 114 patients (7.1%) with AKI and 1501 patients without AKI were included. The baseline characteristics and distribution of AKI stage are shown in Table 1 (76.3% in stage 1, 16.7% in stage 2 and 7.0% in stage 3). A review of the etiology in all 114 patients with AKI revealed that 6 patients had ICI-AKI (5.2% of all AKI patients). Among patients with non-ICI-related AKI, 35 experienced hemodynamic or prerenal AKI and 11 experienced obstructive AKI. The most frequently used ICI in the AKI patient group was nivolumab, and the most common tumor type was hepatobiliary system malignancy (Fig. 2).

Patients with AKI were similar to those without AKI in terms of age, gender, BMI, drug grouping of ICIs, and chemotherapy drugs taken. Compared to patients in the non-AKI group, patients in the AKI group had lower baseline SCr levels and higher baseline eGFRs. Further analysis revealed that although the AKI group had better baseline renal function, physicians gave a greater proportion of potentially nephrotoxic drugs (PPIs, diuretics, NSAIDs, and antibiotics) to patients with better baseline renal function (Table 1).

### Risk factors for AKI in patients receiving ICIs

Univariate logistic analysis showed that baseline eGFR, anemia, baseline albumin (Alb) < 30 g/L, and use of ACEIs/ARBs, antibiotics, diuretics, NSAIDs, PPIs, nivolumab, toripalimab, sintilimab and extrarenal irAEs were associated with the occurrence of AKI (*P* < 0.05). Statistically significant variables from the univariate analysis were included in the multivariate logistic regression analysis, which showed that anemia (OR: 1.95; 95% CI: 1.16–3.28; *P* = 0.0123), baseline Alb < 30 g/L (OR: 1.62; 95% CI: 1.17–2.23; *P* = 0.0034), use of antibiotics (OR: 2.56; 95% CI: 1.10–5.95; *P* = 0.0288), use of diuretics (OR: 1.75; 95% CI: 1.12–2.73; *P* = 0.0132), use of NSAIDs (OR: 3.12; 95% CI: 1.96–4.97; *P* < 0.001), and use of PPIs (OR: 2.07; 95% CI: 1.17–3.66; *P* = 0.0124) were independent risk factors for the development of AKI (Table 2) (Fig. 4).

**Table 2:** Multivariate analysis of risk factors for AKI in patients receiving ICIs.

Risk factors	OR	95% CI	*P* value
eGFR (mL/min·1.73m^2^)	1	0.99–1.01	0.4069
Anemia	1.95	1.16–3.28	0.0123
Alb < 30 g/L	1.62	1.17–2.23	0.0034
30 g/L ≤ Alb ≤ 35 g/L	0.58	0.32–1.09	0.0914
ACEI/ARB	0.25	0.06–1.13	0.0731
Antibiotics	2.56	1.10–5.95	0.0288
Diuretics	1.75	1.12–2.73	0.0132
NSAIDs	3.12	1.96–4.97	< 0.001
PPI	2.07	1.17–3.66	0.0124
Nivolumab	1.20	0.78–1.87	0.4096
Toripalimab	0.44	0.04–5.33	0.5226
Sintilimab	0.53	0.27–1.03	0.0626
Extrarenal irAEs	1.53	0.63–3.72	0.3453

### Baseline characteristics of patients in the nonsurvivor and survivor groups

At the end of the follow-up, a total of 690 patients died (65.8%), and 358 patients survived (34.2%), with a median follow-up time of 804 days (26.8 months) for all patients. The median time from the first ICI drug to death was 417 days (34.75 months) for patients in the nonsurvivor group. A total of 88 patients had AKI (8.4%); 82 patients in the AKI patient group died, with a mortality rate of 93.2%, and 608 patients in the non-AKI group died, with a mortality rate of 63.3%. Compared with the survivor group, the nonsurvivor group had a lower BMI level, a lower proportion of patients using ACEI/ARB drugs (*P* < 0.05), and higher proportions of patients with baseline Alb < 30 g/L, anemia and AKI (*P* < 0.05). There was no significant difference between the two groups in terms of patient age, gender, tumor type, use of chemotherapy drugs, or the proportion of patients classified by the use of ICI drugs (Table 3).

**Table 3:** Univariate analysis of the baseline characteristics of the nonsurvivor group compared to the survivor group during follow-up.

Variable	Cohort	Nonsurvivor	Survivor	*P* value
n	1048	690	358	
Age (years)	58.23 ± 10.93	58.66 ± 11.12	57.41 ± 10.52	0.08
Male, n (%)	728 (69.5)	472 (68.4)	256 (71.5)	0.335
BMI (kg/m^2^)	23.01 ± 3.49	22.62 ± 3.54	23.77 ± 3.26	< 0.001
Baseline creatinine (μmol/L)	70.96 ± 23.46	69.43 ± 24.48	73.90 ± 21.10	0.003
Baseline eGFR (mL/min•1.73m^2^)	94.25 ± 19.28	95.17 ± 19.82	92.48 ± 18.08	0.033
**Baseline Alb, n (%)**				< 0.01
< 30 g/L	46(4.4)	42(6.1)	4(1.1)	
≥30 g/L	1002(95.6)	648(93.9)	354(98.9)	
Malignancy, n (%)				0.064
Digestive system	462 (44.1)	318 (46.1)	144 (40.2)	
Urogenital tract	94 (9.0)	53 (7.7)	41 (11.5)	
Lung	382 (36.5)	253 (36.7)	129 (36.0)	
Other	110 (10.5)	66 (9.6)	44 (12.3)	
**Comorbidities, n (%)**				
Cerebrovascular disease	36 (3.4)	22 (3.2)	14 (3.9)	0.667
CHD	83 (7.9)	51 (7.4)	32 (8.9)	0.448
Diabetes	186 (17.7)	122 (17.7)	64 (17.9)	1
Hypertension	298 (28.4)	191 (27.7)	107 (29.9)	0.497
Anemia	639 (61.0)	485 (70.3)	154 (43.0)	< 0.001
Liver disease	157 (15.0)	95 (13.8)	62 (17.3)	0.151
**Concomitant medications, n (%)**				
ACEI/ARB	141 (13.5)	71 (10.3)	70 (19.6)	< 0.001
PPI	697 (66.5)	473 (68.6)	224 (62.6)	0.061
Diuretics	206 (19.7)	166 (24.1)	40 (11.2)	< 0.001
NSAIDs	412 (39.3)	312 (45.2)	100 (27.9)	< 0.001
Antibiotics	26 (2.5)	22 (3.2)	4 (1.1)	0.067
Chemotherapy	240 (22.9)	145 (21.0)	95 (26.5)	0.052
**Immunotherapy, n (%)**				
Ipilimumab	27 (2.6)	19 (2.8)	8 (2.2)	0.766
Nivolumab	364 (34.7)	274 (39.7)	90 (25.1)	< 0.001
Pembrolizumab	329 (31.4)	213 (30.9)	116 (32.4)	0.662
Durvalumab	7 (0.7)	4 (0.6)	3 (0.8)	0.931
Toripalimab	120 (11.5)	54 (7.8)	66 (18.4)	< 0.001
Sintilimab	241 (23.0)	148 (21.4)	93 (26.0)	0.115
Camrelizumab	12 (1.1)	8 (1.2)	4 (1.1)	1
Atezolizumab	23 (2.2)	15 (2.2)	8 (2.2)	1
**ICIs class, n (%)**				
Anti-PD-1	1015 (96.9)	670 (97.1)	345 (96.4)	0.647
Anti-PD-L1	30 (2.9)	19 (2.8)	11 (3.1)	0.922
Anti-CTLA-4	27 (2.6)	19 (2.8)	8 (2.2)	0.766
Combined immunotherapy	23 (2.2)	17 (2.5)	6 (1.7)	0.546
AKI, n (%)	88 (8.4)	82 (11.9)	6 (1.7)	< 0.001

*ACEI* angiotensin-converting enzyme inhibitor, *ARB* angiotensin receptor blocker, *AKI* acute kidney injury, *eGFR* estimated glomerular filtration rate, *CHD* coronary heart disease, *NSAIDs* nonsteroidal anti-inflammatory drugs, *PPI* proton pump inhibitor, CTLA-4 cytotoxic T lymphocyte–associated antigen 4, PD-1 programmed cell death 1, PD-L1 programmed death-ligand 1, *BMI*, body mass index, *Alb* albumin.

### Risk factors for failure to recover renal function in patients with AKI treated with ICIs

Among 88 patients with AKI, 47 patients (53.4%) failed to recover renal function within 90 days after the occurrence of AKI. Factors such as gender, age, BMI, tumor type, baseline SCr, baseline eGFR, AKI stage, and whether hormone therapy was used after the occurrence of AKI were included in the multivariate logistic regression analysis, and the results showed that the occurrence of stage 2 or 3 AKI (OR: 4.06; 95% CI: 1.15–14.37; *P* = 0.03) was an independent risk factor (Table 4) (Fig. 4).

**Table 4:** Risk factors for nonrecovery of renal function in AKI patients (multivariate logistic regression analysis).

Variable	OR	95% CI	*P* value
Male	2.21	0.28–17.72	0.4537
Age (years)	0.98	0.88–1.08	0.6408
BMI (kg/m^2^)	0.92	0.81–1.06	0.2663
eGFR (mL/min/1.73m^2^)	0.99	0.87–1.13	0.9208
sCr (μmol/L)	0.99	0.89–1.11	0.9166
**Malignancy**			
Urogenital tract	0.66	0.05–9.37	0.7604
Lung	1.05	0.32–3.45	0.9362
Other	0.73	0.17–3.23	0.6801
Stage 2 or 3 AKI	4.06	1.15–14.37	0.03
Treatment with corticosteroids	0.40	0.15–1.04	0.0606

### Risk factors for death in patients treated with ICIs

**Figure 3:**
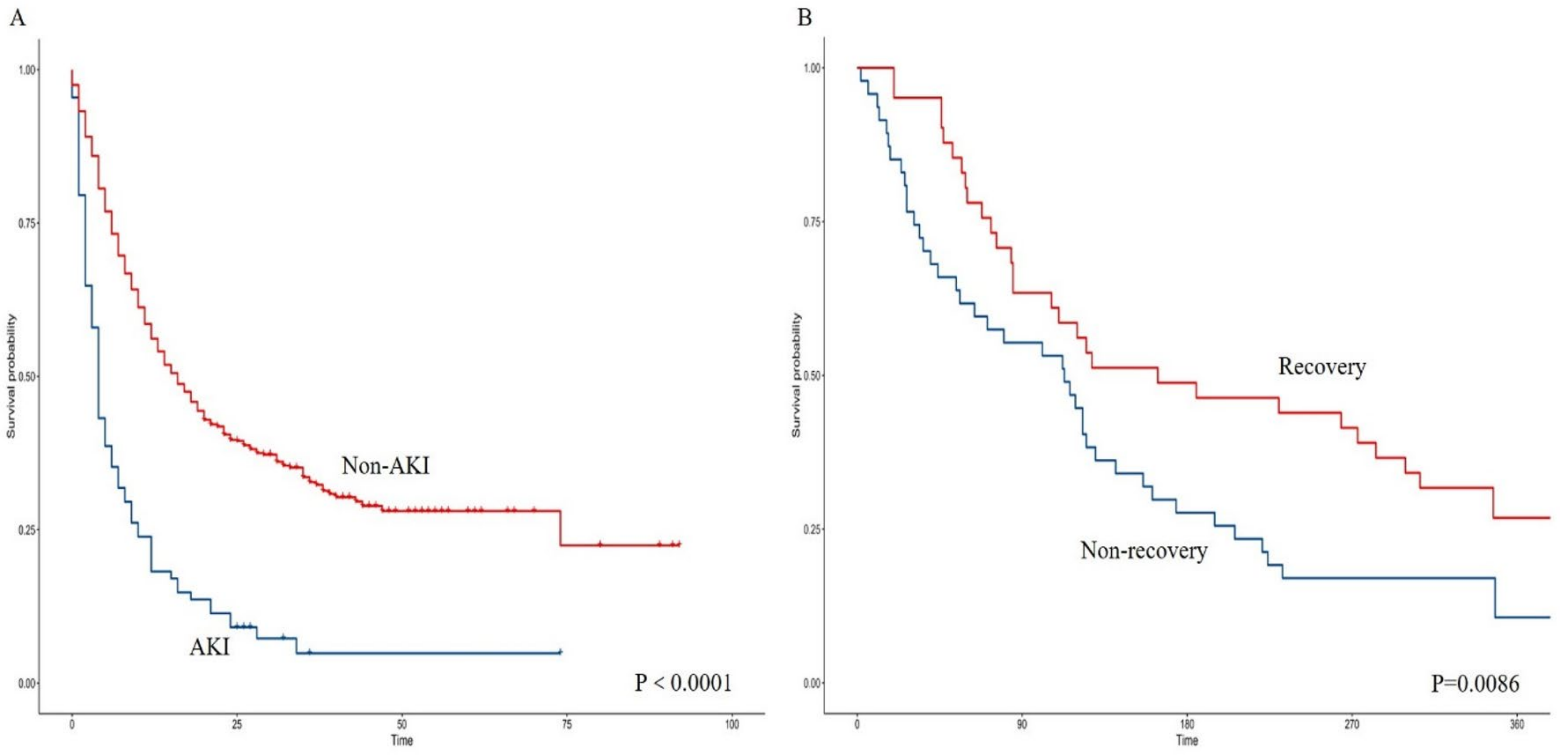
Survival analysis of AKI patients versus non-AKI patients. (**A**). Survival analysis of patients with restored renal function versus those with unrestored renal function. (**B**).

**Figure 4:**
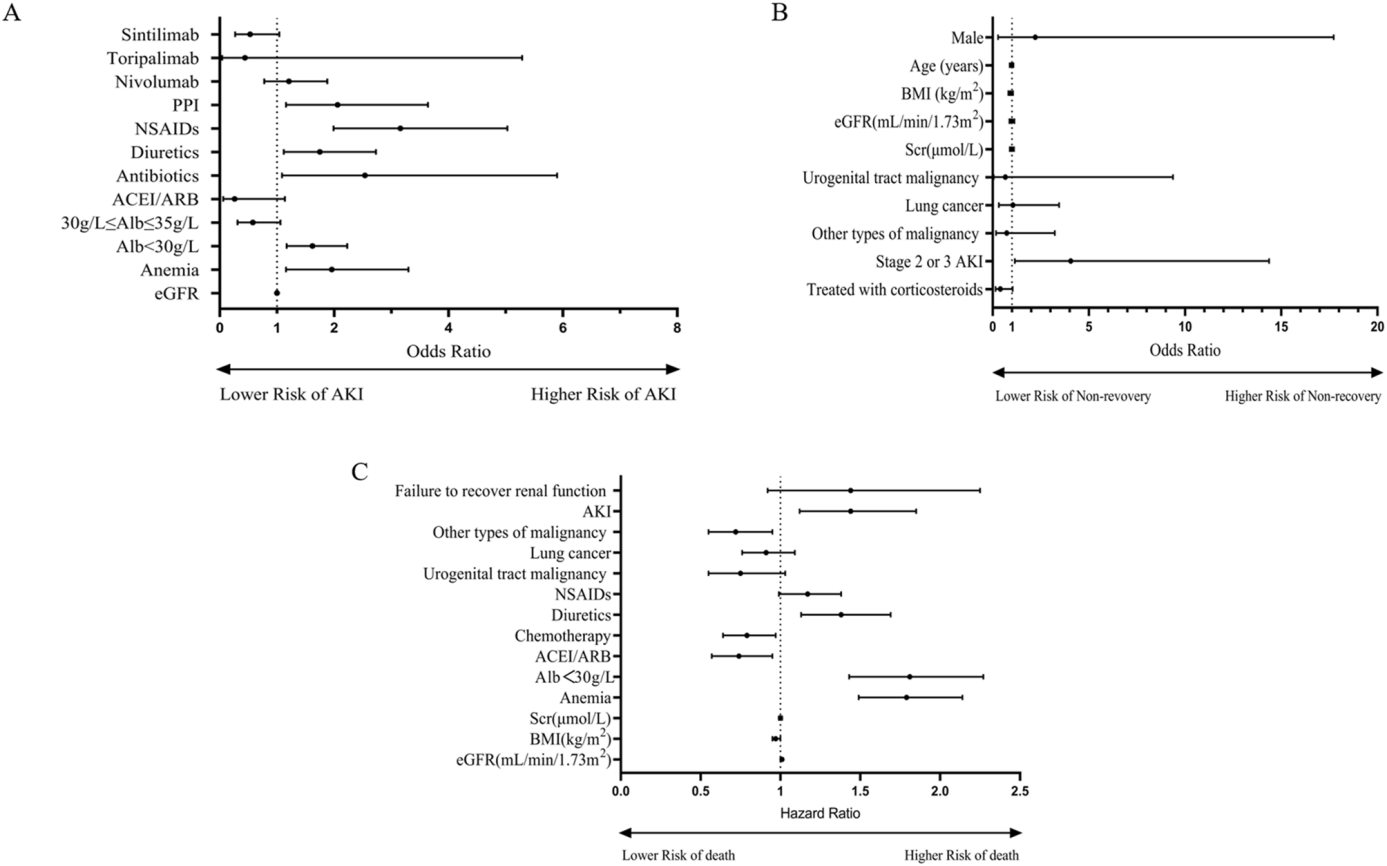
Risk factors for AKI in patients receiving ICIs (**A**). Risk factors for nonrecovery of renal function in AKI patients (**B**). Risk factors for death in patients treated with ICIs (**C**).

Univariate Cox regression analysis showed that eGFR, Alb < 30 g/L, anemia, use of antibiotics, use of NSAIDs, occurrence of AKI, and nonrecovery of renal function after the occurrence of AKI were risk factors for death in patients treated with ICIs (*P* < 0.05) (Fig. 3). Indicators that were statistically significant in the univariate Cox regression analysis were included in the multivariate Cox regression analysis model, and the results of the multivariate Cox regression analysis showed that anemia, Alb < 30 g/L, use of diuretics, and occurrence of AKI were independent risk factors for death in patients treated with ICIs (*P* < 0.05) (Table 5) (Fig. 4); BMI, use of ACEIs/ARBs, use of chemotherapeutic agents, and other types of tumors were protective factors for death in patients treated with ICIs (*P* < 0.05) (Table 5) (Fig. 4).

**Table 5:** Risk factors for death in patients treated with ICIs (multivariate Cox regression analysis).

Variable	HR	95% CI	*P* value
eGFR (mL/min/1.73m^2^)	1.01	1.00–1.01	0.176
BMI (kg/m^2^)	0.97	0.95–1.00	0.027
sCr (μmol/L)	1.00	0.99–1.01	0.978
Anemia	1.79	1.49–2.14	< 0.001
Alb < 30 g/L	1.81	1.43–2.27	< 0.001
ACEI/ARB	0.74	0.57–0.95	0.017
Chemotherapy	0.79	0.64–0.97	0.025
Diuretics	1.38	1.13–1.69	0.001
NSAIDs	1.17	0.99–1.38	0.060
**Malignancy**			
Urogenital tract	0.75	0.55–1.03	0.073
Lung	0.91	0.76–1.09	0.304
Other	0.72	0.55–0.95	0.021
AKI	1.44	1.12–1.85	0.004
Failure to recover renal function	1.44	0.92–2.25	0.109

## Discussion

ICIs are new anticancer drugs that have revolutionized the natural course of many malignancies, such as melanoma, renal cell carcinoma, non-small-cell lung cancer, bladder cancer, Hodgkin's lymphoma and other malignancies^31^. Although ICIs have greatly improved the prognosis of patients with malignancies, a number of irAEs have also occurred, one of which is AKI. Initially, the nephrotoxicity of ICIs was often referred to as "creatinine elevation" in some oncology clinical trials^32^ and was not further investigated. In recent years, however, there has been growing evidence that the occurrence of AKI has a significant impact on the prognosis of patients treated with ICIs.

This is one of the largest published series evaluating AKI in more than 1600 patients treated with ICIs at a single center. First, AKI was found to be a common complication in patients treated with ICIs in this study, which showed an incidence of 7.1% for AKI. In this study, anemia, baseline Alb < 30 g/L, and the use of antibiotics, diuretics, NSAIDs, and PPIs were found to be independent risk factors for developing AKI in patients treated with ICIs. Second, we followed up patients and found that the risk of death was higher in patients who developed AKI with unrecoverable renal function than in patients with recovered renal function and that the development of stage 2 or 3 AKI was an independent risk factor for unrecoverable renal function after the development of AKI in patients treated with ICIs. Third, further studies found that baseline anemia, baseline Alb < 30 g/L, occurrence of AKI, and use of diuretics were independent risk factors for death in patients; higher baseline BMI, other tumor types, use of ACEIs/ARBs, and use of chemotherapeutic agents were protective factors for patient death.

Univariate and multivariate logistic regression analyses revealed that the use of NSAIDs, antibiotics, and PPIs were independent risk factors for the development of ICI-AKI. This is consistent with the findings of Harish Seethapathy^27^, Maen Abdelrahim^33^ and Frank B Cortazar^25^ et al. Their studies all showed the use of PPIs as a risk factor for ICI-AKI. First, PPIs, NSAIDs and antibiotics can cause acute tubulointerstitial nephritis (ATIN), and the mechanism by which these 3 drugs cause ATIN may be related to their resulting drug-specific T-cell activation in hypersensitivity reactions^34,35^. Second, treatment with ICIs may lead to loss of tolerance to potentially nephrotoxic drugs by reactivating drug-specific T cells in some patients, lowering the body's tolerance threshold to potentially nephrotoxic drugs^36^. In our study, we did not find extrarenal irAEs to be associated with the occurrence of AKI by multivariate logistic regression analysis, which differs from the results of previous studies^19,30^; this difference may be related to the low number of patients with irAEs in our center and possibly to the lack of diagnosis or course of disease documentation for minor irAEs by clinicians; in addition, our study for extrarenal irAEs was concomitant with the occurrence of AKI, which may have contributed to this result.

In this study, 690 patient deaths were recorded at the end of the follow-up, with a very high mortality rate (65.8%), which is similar to the findings of Claire Stein^37^, Meraz-Muñoz^38^, and Clara et al^23^. The mortality rates of patients in their studies ranged from 52.3 to 72.0%. The higher patient mortality results may be attributed to the characteristics of the oncology department in our center, where some patients with refractory malignancies nationwide are referred to our center for treatment because of the high number of RCT studies conducted in our oncology department; on the other hand, ICIs are often used to treat many patients with metastatic and refractory malignancies^39^, which may also contribute to the higher patient mortality rate at the end of the follow-up of this study. The results of this study showed that the occurrence of AKI was an independent risk factor for death in patients treated with ICIs, which is consistent with the findings of Garcıá-Carro^23^ and Shruti Gupta^24^ et al. There are two possible reasons for this result. First, the general condition of patients with AKI is poorer. In our study, the proportions of patients with baseline Alb < 30 g/L and anemia were higher in the AKI group than in the non-AKI group, and both baseline Alb < 30 g/L and anemia were found to be independent risk factors for AKI and death. Lower baseline anemia and lower baseline Alb levels may, to some extent, be markers of poor baseline basal status; second, AKI leads to deterioration of the overall condition of patients, causing disturbance of the internal environment through various pathophysiological mechanisms, which leads to death.

In this study, we studied a subgroup of patients who developed AKI. Forty-seven patients (53.4%) developed AKI and then failed to recover renal function. The development of stage 2 or 3 AKI was an independent risk factor for failure to recover renal function in patients with AKI, which also reflects the importance of the early identification and diagnosis of AKI and aggressive treatment to prevent the progression of stage 1 AKI to stage 2 or 3 AKI. In survival analysis, the risk of death was found to be greater in patients who failed to recover renal function within 90 days after the onset of AKI than in those who recovered renal function, which is consistent with the findings of Cortazar, F. B^25^ et al. The increased risk of death due to failure to recover renal function after the occurrence of AKI may be because oncologists have very limited options for cancer treatment in some patients with persistent renal impairment, which in turn leads to tumor progression and an increased risk of death; therefore, this reflects the importance of the early recognition and treatment of this adverse event before irreversible renal damage occurs.

It should be specifically noted that the majority of patients included in this study had normal renal function at baseline. This may be because many patients participated in clinical trials at our institution and because many therapeutic trials of cancer drugs exclude patients with CKD and ESRD. Therefore, future studies need to focus more on this group of patients and provide more research data to address the development of ICI-AKI in the CKD population and its impact on kidney prognosis and cancer prognosis. This may become increasingly important as many cancer patients with CKD will become candidates for immunotherapy as the indications for immunologic agents expand.

Although this is the largest study of ICI-AKI conducted in Chinese patients, we acknowledge some limitations. First, not all patients with ICI-AKI in this study underwent renal puncture biopsy and nephrology laboratory tests, such as routine urine tests, so it was not possible to accurately distinguish the specific etiology of AKI. Second, another limitation of this study is that the cause of death of the patients who died was not considered. Third, our outcome analysis focused on renal recovery and overall survival, and no data were collected on tumor response after patients were treated with ICIs. Finally, this study was a single-center retrospective study, and some bias may exist in the analysis.

In conclusion, this study showed that the incidence of ICI-AKI was 7.1%. Anemia, baseline Alb < 30 g/L, and the use of antibiotics, diuretics, NSAIDs, and PPIs were independent risk factors for the development of AKI in patients receiving ICIs. The risk of death was higher in patients with unrecoverable renal function after the occurrence of ICI-AKI than in patients with recovered renal function, and the occurrence of high-grade AKI (stage 2 or 3 AKI) was an independent risk factor for the occurrence of unrecoverable renal function after AKI in patients treated with ICIs. Baseline anemia, baseline Alb < 30 g/L, occurrence of acute kidney injury, and use of diuretics were independent risk factors for death in patients treated with ICIs. Higher baseline BMI, other types of tumors, use of ACEIs/ARBs, and use of chemotherapeutic agents were protective factors against death in patients treated with ICIs.

## Data Availability

The datasets generated and analysed during the current study are not publicly available due regulations on the Big Data Research Center of the Chinese PLA General Hospital but are available from the corresponding author on reasonable request.
